# S_6_N_2_O_15_—A Nitrogen‐Poor Sulfur Nitride Oxide, and the Anhydride of Nitrido‐*tris*‐Sulfuric Acid

**DOI:** 10.1002/anie.202005056

**Published:** 2020-08-04

**Authors:** David van Gerven, Mathias S. Wickleder

**Affiliations:** ^1^ University of Cologne Institute of Inorganic Chemistry Greinstr. 6 50939 Cologne Germany

**Keywords:** molecular cages, Sulfur nitride oxide, sulfur trioxide, sulfuric acid derivatives

## Abstract

The reaction of hexachlorophosphazene, P_3_N_3_Cl_6_, with SO_3_ leads to the new sulfur nitride oxide S_6_N_2_O_15_. The compound displays an extraordinarily low nitrogen content and exhibits a bicyclic cage structure according to the formulation N{S(O)_2_O(O)_2_S}_3_N, with both nitrogen atoms in trigonal planar coordination of sulfur atoms. Interestingly, the new nitride oxide can be also seen as the anhydride of nitrido‐*tris*‐sulfuric acid, N(SO_3_H)_3_.

## Graphical abstract

**The cage type molecule** S_6_N_2_O_15_ is not only a new sulfur nitride oxide, but also the anhydride of the rarely seen nitrido‐*tris*‐sulfuric acid. Interestingly, the unique compound is obtained in high yields from reactions of SO_3_ with P_3_N_3_Cl_6_.

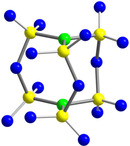

Sulfur trioxide, SO_3_, is an interesting reagent in chemical reactions. On one hand, it is a very strong oxidizer and on the other hand, it can act as a typical Lewis base. We have used the oxidation strength of SO_3_, especially under harsh conditions, for the oxidation of noble metals and noble metal compounds. The formation of two modifications of Pd(S_2_O_7_) by the reaction of elemental palladium with SO_3_ is a nice example of these efforts.^1^, ^2^ On the other hand, SO_3_ is a strong Lewis acid and forms readily adducts with several Lewis bases. Well known examples are the complexes with dioxane and pyridine (py).^3^, ^4^ Of the latter, SO_3_⋅py, is even a commercial product that serves as a safe SO_3_ source for many reactions. In fact, Lewis acid/base adducts with N‐donor molecules and SO_3_ have been studied quite extensively starting already in the 1950s.^5^ Actually, very spectacular compounds have been prepared at that time, for example, the adducts S_4_N_4_⋅*x* SO_3_ (*x=*1–4), for which S_4_N_4_⋅SO_3_ was structurally characterized later.^6^ Another potential base that has been considered for SO_3_ interaction was hexachlorophosphazene, P_3_N_3_Cl_6_.^5^ It has formally three available nitrogen atoms bearing free electron pairs. Thus, the composition P_3_N_3_Cl_6_⋅3 SO_3_ of the reported complex it is very reasonable, even if structural information is still lacking. We came across that compound for two reasons: On one hand, we are interested in Lewis acid/base complexes of SO_3_ since we discovered that the rarely known polysulfates [S_*n*_O_3*n*+1_]^2−^ can be described as adducts according to [S_*n*_O_3*n*+1_]^2−^⋅SO_3_, at least for larger numbers of *n*.^7^, ^8^ In the hexasulfate Rb_2_[S_6_O_19_] (*n=*6), the distance of the sulfur atom of SO_3_ to the next oxygen atom is already as long as 231 pm.^8^ For a detailed investigation of bond lengths within Lewis acid/base complexes structure elucidations of complexes with different bases are desirable. On the other hand, we have recently started a research project aiming at a detailed understanding of nitrogen‐based derivatives of sulfuric acid. These are for example the slightly acidic sulfimide, SO_2_(NH_2_)_2_,^9^ and its cyclic condensation products S_3_O_6_(NH)_3_ and S_4_O_8_(NH)_4_,^10^, ^11^ for which a limited number of salts are known.^12^, ^13^, ^14^ However, the more prominent of these derivatives are amidosulfuric acid, imido‐*bis*‐sulfuric acid, and nitrido‐*tris*‐sulfuric acid (Figure 1). Even if all of these acids are textbook examples, our knowledge is still quite limited. Only amidosulfuric acid, in its zwitterionic ground state a Lewis acid/base complex of SO_3_ and NH_3_, and amidosulfates have been frequently reported.^15^ For all of the other anions depicted in Figure 1 a very limited number of salts is known.^16^ Especially for the nitrido‐*tris*‐sulfuric acid, N(SO_3_H)_3_, which is not known in a pure form, there is just one report of respective salt, namely K_3_[N(SO_3_)_3_]⋅2 H_2_O.^17^ The acid and their salts are prone to hydrolysis, what is certainly a drawback for synthesis, especially from aqueous solution. In this case, hexachlorophosphazene might be a suitable nitrogen source for the preparation of N‐based sulfuric acids under anhydrous conditions.

**Figure 1:**
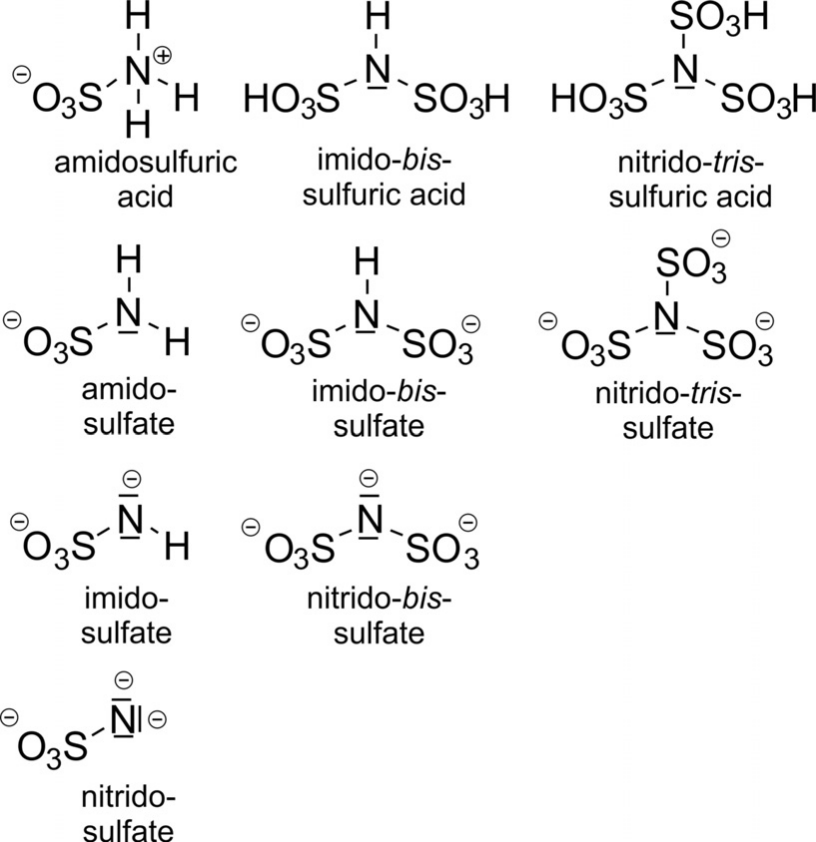
Nitrogen‐based sulfuric acid derivatives.

With respect to the two above‐mentioned issues, that is, hexachlorophosphazene as Lewis base and as starting material for the synthesis of N‐based sulfuric acid derivates, we have investigated the reaction of SO_3_ and P_3_N_3_Cl_6_ under various conditions in more detail. According to the findings of Goehring et al., at low temperature (ca. 40 °C) a reaction is observed, however without gaining crystalline material. Only if the temperature is raised to 80 °C a huge number of single crystals grow from excess SO_3_ in a short time (Figure 2). Structure elucidation revealed that the anhydride of nitrido‐*tris*‐sulfuric acid had formed, namely S_6_N_2_O_15_. With respect to the amount of the gained product, the reaction is almost quantitative, so that the reaction could be written as 2 P_3_N_3_Cl_6_+18 SO_3_→3 S_6_N_2_O_15_+4 POCl_3_+1/2 P_4_O_10_. We have not identified the by‐products unambiguously up to now, however, we do not observe elemental chlorine, which is, according to Ref. 5, a reaction product at higher temperature. A very likely product is phosphoryl chloride, POCl_3_. The presence of POCl_3_ would also explain that the sulfur trioxide which is used in excess in the reaction stays liquid, even if the ampoules are stored in a refrigerator. In similar reactions, we usually observe the formation of asbestos type sulphur trioxide (α‐SO_3_) at lower temperature, visible by large needle shaped crystals growing in the ampoule. Compounds such as SO_2_Cl_2_ or POCl_3_ are well‐known stabilizers that are used to keep sulfur trioxide liquid below 30 °C by supressing the polymerisation of SO_3_ molecules.^18^, ^19^ Attempts to separate the obtained by‐product from SO_3_ failed up to now.

**Figure 2:**
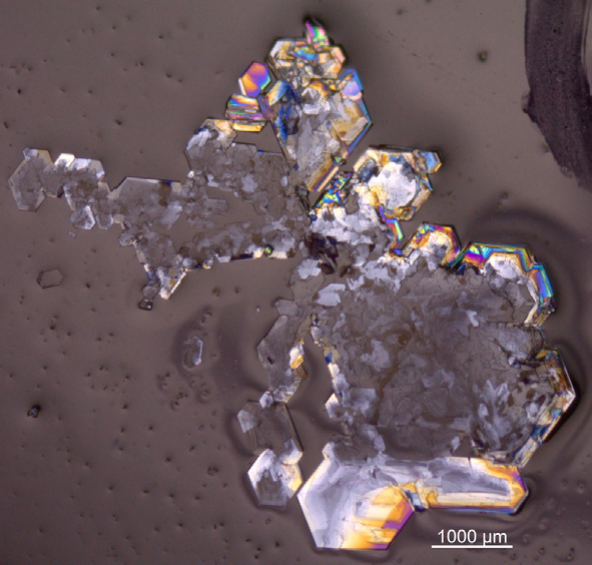
Single crystals of S_6_N_2_O_15_.

The molecular compound has a unique structure with two three‐coordinate nitrogen atoms connected by three [S_2_O_5_] groups (Figure 3), as it would be emphasized by the more descriptive formula N{S(O)_2_O(O)_2_S}_3_N_._ The distances S–N fall is a narrow range between 170.6 and 171.6 pm, and the surrounding of the nitrogen atoms is almost perfectly planar. Thus, no activity of the lone electron pair is observable, obviously due to significant π‐bonding to the sulfur atoms. The observation is in line with the reported findings for the anion [N(SO_3_)_3_]^3−^.^17^ The nitrogen atoms are connected by three nearly identical S‐O‐S bridges, displaying distances 161.8 and 163.8 pm and angles S‐O‐S of about 125°. These are the typical values that are, for example, observed for the disulfate ion, S_2_O_7_^2−^. The distances and angles within the S_6_N_2_O_15_ molecule are well reflected by quantum mechanical calculations (cf. caption Figure 3 and Supporting Inforamtion). As expected, the calculations result in *C*_3*h*_ symmetry for the molecule, while in the solid state (space group *C*2/*c*) only *C*_1_ symmetry is found.

**Figure 3:**
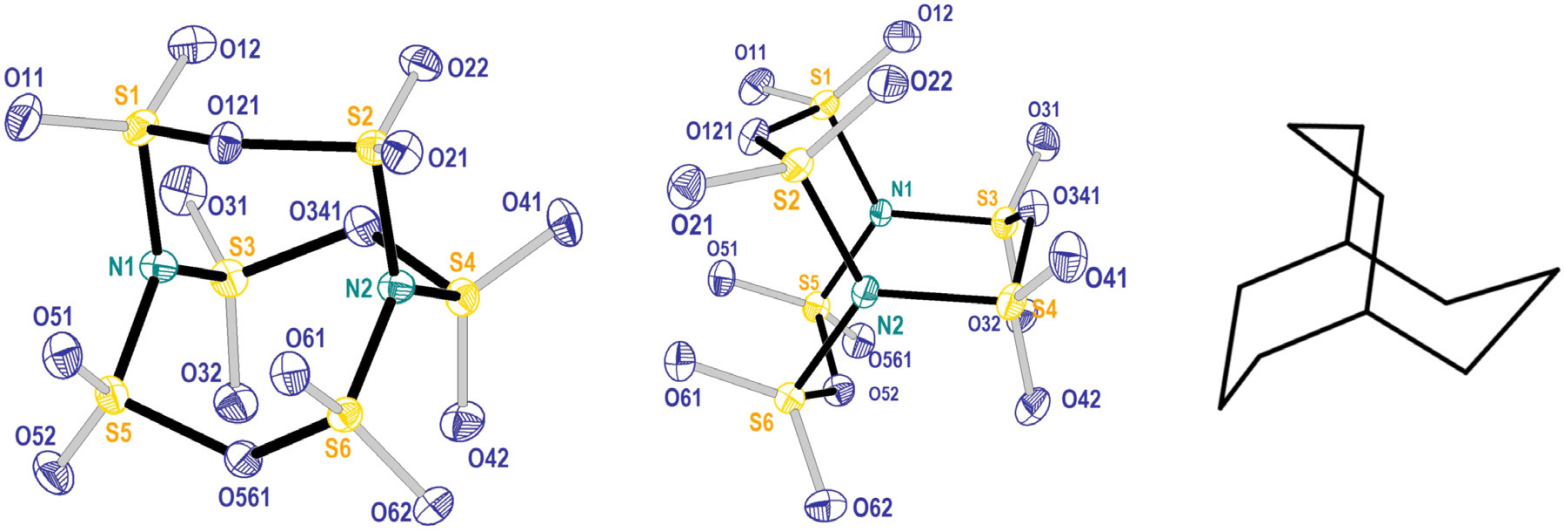
Structure and labelling of the S_6_N_2_O_15_ molecule viewed in different directions. The middle picture shows the molecule viewed along an axis through the nitrogen atoms, emphasizing their almost perfect trigonal planar coordination by sulfur atom. At right, the molecule bicyclo[3.3.3]undecane is depicted which represents the [S_6_O_3_N_2_] cage of S_6_N_2_O_15_ (emphasized by black bonds). Selected distances (in pm) and the theoretical values (in *italics*): S(1‐6)‐O_terminal_ (O11, O12; O21, O22; O31, O32; O41, O42; O51, O52; O61, O62) ca. 140.5(2)/*141.75*, S1‐O121 161.8(1)/*164.12*, S2‐O121 163.8(2)/*164.2*, S3‐O341 163.6(2)/*164.14*, S4‐O341 162.3(2)/*164.12*, S5‐O561 163.1(2)/*164.13*, S6‐O561 162.8(2)/*164.13*, N1‐S1 170.6(2)/*172.64*; N1‐S3 171.6(2)/*172.67*, N1‐S5 171.6(2)/*172.67*, N2‐S2 171.5(2)/*172.67*, N2‐S4 171.6(2)/1*72.69*, N2‐S6 171.6(2)/*172.69*.^31^

The core cage of the S_6_N_2_O_15_ molecule (emphasized by black bonds in Figure 3) has the shape of the bicyclic organic molecule bicyclo[3.3.3]undecane. Such a cage has not been observed before in the chemistry of sulfur nitride‐oxides, although a significant number of compounds has been observed in the system S/N/O (Figure 4).^20^ With respect to the structural characterizations these compounds show chain structures, such as S_2_(NSO)_2_ 21 or S_3_N_2_O_2_,^22^, ^23^ cyclic molecules like S_3_N_2_O_5_,^24^, ^25^ S_7_N_6_O_8_,^26^ and S_4_N_4_O_2_,^27^ as well as ionic species such as (NO)_2_[S_4_O_13_].^28^ The most unusual compound among the molecular sulfur nitride oxides is probably the adduct S_4_N_4_⋅SO_3_ which has already been mentioned in the introduction.^6^ Another outstanding molecule is sulfuryl azide, SO_2_(N_3_)_2_,^29^ which is the nitrogen richest molecule in the S/N/O system. The new compound S_6_N_2_O_15_ is up to now the sulfur nitride oxide with the highest oxygen and the lowest nitrogen content.

**Figure 4:**
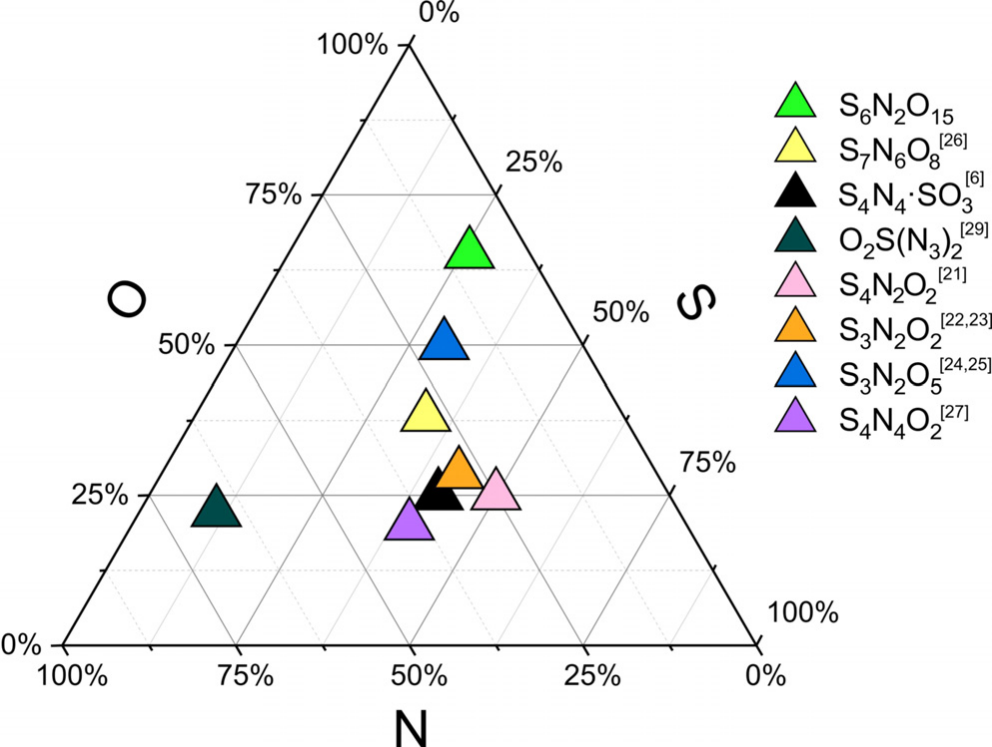
Molecular compounds in the system S/N/O according to the atomic ratios.

The successful synthesis of S_6_N_2_O_15_ by the reaction of P_3_N_3_Cl_6_ and SO_3_ leads to several new directions that are worth pursuing. On one hand, the reaction might also be suitable for the preparation of the rarely seen nitrido‐*tris*‐sulfates, if suitable cations are added to the reaction mixture. On the other hand, variation of the reaction conditions may lead to other species is thinkable, for example the [N(SO_3_)_2_]^3−^ ion mentioned in the introduction (cf. Figure 1). Moreover, even anions with both, tri‐ and bi‐coordinate nitrogen atoms come into sight, for example, the hypothetical anion [S_6_N_3_O_12_]^3−^. Finally, it is worth remembering that there is no nitrido sulfate ion, [SN_4_]^6−^, known up to now, also not in the form of condensed species. This finding for sulfur is in strong contrast to the findings for the neighboring elements silicon and phosphorous. Only in organic derivatives, such as the famous [S(N^t^Bu)_4_]^2−^ ion, is a complete nitrogen coordination possible so far.^30^

## Conflict of interest

The authors declare no conflict of interest.

## Supporting information

As a service to our authors and readers, this journal provides supporting information supplied by the authors. Such materials are peer reviewed and may be re‐organized for online delivery, but are not copy‐edited or typeset. Technical support issues arising from supporting information (other than missing files) should be addressed to the authors.

Supplementary
