# Supplementary material for: S6N2O15—A Nitrogen‐Poor Sulfur Nitride Oxide, and the Anhydride of Nitrido‐tris‐Sulfuric Acid
Source: Angew Chem Int Ed Engl. 2020 Aug 4;59(39):17169–71. doi: 10.1002/anie.202005056 (PMC7540367; doi:10.1002/anie.202005056)
Supplement: Supplementary file 1 — Supplementary [file ANIE-59-17169-s001.pdf]

Angewandte Chemie
Eine Zeitschrift der Gesellschaft Deutscher Chemiker

Supporting Information

$S_6N_2O_{15}$—A Nitrogen-Poor Sulfur Nitride Oxide, and the Anhydride of Nitrido-*tris*-Sulfuric Acid

*David van Gerven and Mathias S. Wickleder**

anie_202005056_sm_miscellaneous_information.pdf

SUPPORTING INFORMATION

Abstract: The reaction of hexachlorophosphazene, $P_3N_3Cl_6$, with SO_3 leads to the new sulfur nitride-oxide $S_6N_2O_{15}$. The compound displays an extraordinarily low nitrogen content and exhibits a bicyclic cage structure with both nitrogen atoms in trigonal planar coordination of sulfur atoms. Interestingly, the new nitride-oxide can be also seen as the anhydride of nitrido-*tris*-sulfuric acid, $N(SO_3H)_3$.

DOI: 10.1002/anie.2016XXXXX

Table of Contents

A. Synthesis
B. Structure determination and crystallographic details
C. Results from quantum chemical calculations
D. Vibrational spectroscopy
E. Powder XRD

((Include the title of each section along with its page or section number.))

A. Synthesis

SO_3 was obtained in a specially designed apparatus for the generation, distillation and the subsequent transfer into glass ampoules under nitrogen gas. For this purpose, fuming sulfuric acid (5 mL, 65 % SO_3, used as received, Merck, Darmstadt, Germany) was slowly added via a dropping funnel into a 500 mL flask with P_4O_{10} (250 g, >97 %, Merck, Darmstadt). At the same time, the flask was heated at 130 °C and the generated SO_3 distilled into a connected burette body (scaling 0.01 mL ± 0.01). A connected glass ampoule (*l* = 200 mm, *ø* = 20 mm, thickness of the tube wall = 2 mm) containing $P_3N_3Cl_6$ (150 mg, 0.431 mmol) was then filled with 1.92 g (24.0 mmol) of freshly distilled SO_3. The ampoule was torch sealed and heated to 80 °C in a tube furnace. This temperature was maintained for 48 h and the reaction mixture was then allowed to cool down to room temperature within 100 h. $S_6N_2O_{15}$ could be obtained in form of transparent highly moisture sensitive crystals, which have to be handled under inert gas conditions.
Caution! SO_3 is a strong oxidizer which needs careful handling. During the reaction and even after cooling down to room temperature the glass tube might be under pressure. The tube should be cooled with liquid nitrogen before opened.

B. Structure determination and crystallographic details

Crystal structure determination has been performed at the P24.1 beamline of the PETRA III facility at German Electron Synchrotron (DESY) Hamburg (Germany). Therefore, single crystals of $S_6N_2O_{15}$ were prepared under inert oil and selected with the aid of a polarization filter of a light microscope. Attached to a micromount, one single crystal was positioned in the cold nitrogen gas stream (100.0(2) K) of the single crystal diffractometer (Huber 4-circle Kappa, P24 Beamline, Petra III) and intensity data was collected. Both the collect intensity data were reduced, and a cell refinement was carried out.[1] The structure solution under the assumption of the respective space group was successful by intrinsic phasing (SHELXT).[2] Finally, anisotropic displacement parameters were introduced and a multi-scan absorption correction was applied to the reflection data. Atomic positions and further details of the crystal structures can be obtained from the joint CCDC/FIZ Karlsruhe deposition service on quoting the deposition number given in Table S1.

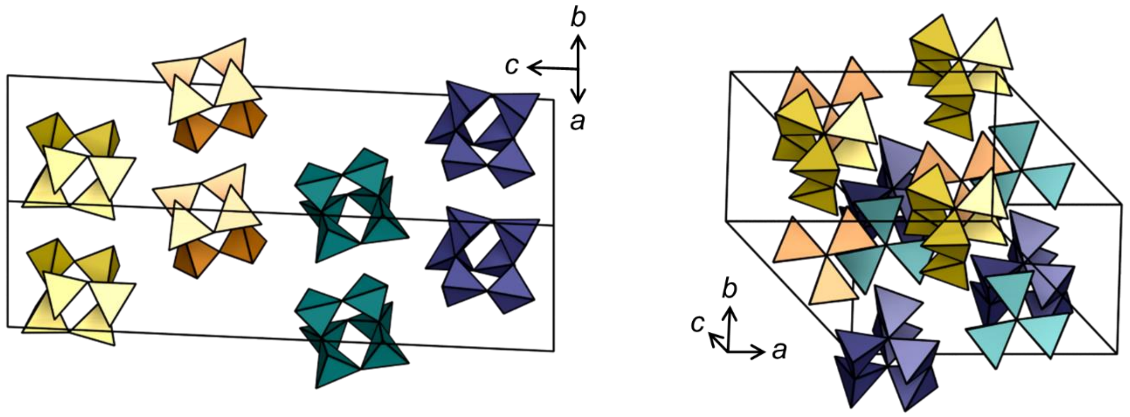

Figure S2. Crystal structure of $S_6N_2O_{15}$ viewed along two non-defined spatial directions. The sulfate tetrahedra of the crystallographically indistinguishable molecules are separated by colour for clarity.

Table S1. Crystal data and structure refinement for $S_6N_2O_{15}$.

Empirical formula	$S_6N_2O_{15}$	
Formula weight	460.38	
Temperature	100.0(2) K	
Wavelength	47.686 pm (synchrotron radiation)	
Crystal system	monoclinic	
Space group	*C*2/c	
Unit cell dimensions	a = 1243.70(4) pm	α = 90°
	b = 711.61(2) pm	β = 95.148(1)°
	c = 2696.4(1) pm	γ = 90°
Volume	2.3768(1) nm^3	
Z	8	
Density (calculated)	2.573 g · cm^{-3}	
Absorption coefficient	4.019 cm^{-1}	
F(000)	1840.0	
Crystal size	0.2 × 0.2 × 0.2 mm	
2Theta range for data collection	2,034 to 45,998	
Index ranges	-19 ≤ h ≤ 19, -10 ≤ k ≤ 11, -36 ≤ l ≤ 36	
Reflections collected	26172	
Independent reflections	4594 [*R(*int) = 0.0405]	
Completeness to theta = 90	100%	
Absorption correction	Multi-Scan	
Max. and min. transmission	0.923 and 0.923	
Refinement method	intrinsic	
Data / restraints / parameters	4594/0/209	
Goodness-of-fit on F^2	1.166	
Final R indices [*I*>2sigma(*I*)]	R_1 = 0.0378, wR_2 = 0.1049	
R indices (all data)	R_1 = 0.0418, wR_2 = 0.1064	
Extinction coefficient	0.0069(6)	
Largest diff. peak and hole	0.74 /-0.63	
CCDC number	1901458	

Table S2. Atomic coordinates (× 10^4) and equivalent isotropic displacement parameters (pm^2 × 10^7) for $S_6N_2O_{15}$. *U(eq)* is defined as one third of the trace of the orthogonalized U^{ij} tensor.

Atom	*x*	*y*	*z*	*U(eq)*
N1	3253(1)	3512(2)	4257.4(6)	9.3(3)
N2	2737(1)	4454(2)	3197.0(6)	9.6(3)
O11	1547(1)	2488(2)	4647.0(6)	15.2(3)
O12	2459(1)	358(2)	4084.9(6)	14.7(3)
O121	1394(1)	3154(2)	3779.2(5)	11.0(2)
O21	712(1)	4044(2)	2956.9(6)	13.1(3)
O22	1939(1)	1268(2)	3061.6(6)	13.1(3)
O31	4626(1)	832(2)	4352.6(6)	16.1(3)
O32	5188(1)	4076(2)	4142.2(6)	13.8(3)
O341	4129(1)	2005(2)	3532.4(5)	11.4(2)
O41	3795(1)	2328(2)	2651.9(6)	14.5(3)
O42	4702(1)	4953(2)	3131.9(6)	13.9(3)
O51	2105(1)	6193(2)	4484.9(6)	13.9(3)
O52	4078(1)	6344(2)	4737.3(6)	14.9(3)
O561	3477(1)	6750(2)	3876.9(5)	11.2(2)
O61	1604(1)	7075(2)	3470.0(6)	12.7(2)
O62	3244(1)	7869(2)	3042.4(6)	13.5(3)
S1	2121.5(4)	2146.1(7)	4230.7(2)	10.3(1)
S2	1603.9(4)	3078.8(6)	3188.6(2)	9.52(9)
S3	4456.6(4)	2569.4(6)	4114.5(2)	10.5(1)
S4	3948.3(4)	3482.9(7)	3073.9(2)	10.2(1)
S5	3189.5(4)	5852.1(6)	4405.5(2)	10.3(1)
S6	2677.5(4)	6775.3(6)	3364.7(2)	9.58(9)

Table S3. Anisotropic Displacement Parameters (pm^2 × 10^7) for $S_6N_2O_{15}$. The Anisotropic displacement factor exponent takes the form: $-2\pi^2$ [$h^2 a^{*2} U_{11}$ + 2 h k a* b* U_{12} +…].

**Atom**	U_{11}	U_{22}	U_{33}	U_{23}	U_{13}	U_{12}
N1	6.8(6)	9.1(6)	12.3(7)	-0.9(5)	1.8(5)	0.7(5)
N2	7.3(6)	9.6(6)	12.2(7)	-0.9(5)	2.6(5)	0.1(5)
O11	12.9(6)	22.2(7)	11.0(7)	2.5(5)	2.9(5)	-2.2(5)
O12	13.9(6)	12.0(6)	18.1(7)	1.1(5)	0.1(5)	-1.5(5)
O121	8.0(5)	15.5(6)	9.5(6)	1.0(4)	1.2(4)	0.4(4)
O21	9.8(6)	16.4(6)	12.7(7)	1.4(5)	-1.3(5)	0.6(5)
O22	12.5(6)	12.0(6)	14.8(7)	-3.1(4)	0.6(5)	-1.9(5)
O31	14.2(6)	16.8(7)	17.0(7)	2.7(5)	-0.8(5)	4.5(5)
O32	8.3(5)	17.2(6)	15.8(7)	-2.2(5)	0.4(5)	-2.2(5)
O341	10.5(5)	12.3(6)	11.4(6)	-1.2(4)	0.8(4)	1.8(5)
O41	14.6(6)	18.0(6)	11.0(6)	-4.7(5)	2.4(5)	2.1(5)
O42	8.6(5)	16.4(6)	17.2(7)	0.2(5)	3.3(5)	-1.9(5)
O51	11.7(6)	16.3(6)	14.3(7)	-2.0(5)	4.3(5)	2.8(5)
O52	14.0(6)	18.1(7)	12.2(7)	-4.0(5)	-1.7(5)	-1.8(5)
O561	10.5(5)	12.1(6)	10.8(6)	-0.2(4)	-0.3(4)	-0.8(4)
O61	10.6(6)	14.2(6)	13.4(7)	-0.7(4)	1.9(5)	2.6(5)
O62	15.3(6)	12.4(6)	13.0(7)	2.9(4)	2.9(5)	-1.9(5)
S1	8.5(2)	12.2(2)	10.1(2)	1.6(1)	1.2(1)	-1.2(1)
S2	7.8(2)	11.3(2)	9.4(2)	-0.4(1)	0.3(1)	-1.0(1)
S3	7.6(2)	12.3(2)	11.3(2)	-0.4(1)	-0.0(1)	0.9(1)
S4	8.0(2)	12.8(2)	10.2(2)	-1.6(1)	2.5(1)	-0.1(1)
S5	9.9(2)	12.0(2)	9.1(2)	-1.7(1)	1.1(1)	0.2(1)
S6	9.1(2)	9.5(2)	10.2(2)	0.4(1)	1.5(1)	0.2(1)

Table S4. Experimental bond lengths for $S_6N_2O_{15}$ (in pm).

Atom	Atom	Length	Atom	Atom	Length
S1	O11	140.5(2)	S1	O121	161.8(1)
S1	O12	140.7(2)	S2	O121	163.8(2)
S2	O21	140.3(2)	S3	O341	163.6(2)
S2	O22	140.6(2)	S4	O341	162.3(2)
S3	O31	140.0(2)	S5	O561	163.1(2)
S3	O32	140.4(2)	S6	O561	162.8(2)
S4	O41	140.3(2)	N1	S1	170.6(2)
S4	O42	140.4(2)	N1	S3	171.6(2)
S5	O51	140.6(2)	N1	S5	171.6(2)
S5	O52	140.3(2)	N2	S2	171.5(2)
S6	O61	140.6(1)	N2	S4	171.6(2)
S6	O62	140.2(2)	N2	S6	171.6(2)

Table S5. Experimental bond angles [°] for $S_6N_2O_{15}$.

Atom	Atom	Atom	Angle	Atom	Atom	Atom	Angle
S3	N1	S5	119.40(9)	O31	S3	N1	109.90(9)
S1	N1	S3	120.05(9)	O32	S3	O341	110.06(9)
S1	N1	S5	120.52(9)	O31	S3	O32	125.4(1)
S2	N2	S6	119.86(9)	O341	S3	N1	99.47(8)
S6	N2	S4	119.89(9)	O341	S4	N2	100.25(8)
S2	N2	S4	120.14(9)	O41	S4	O341	103.75(9)
S1	O121	S2	126.15(9)	O42	S4	N2	105.48(8)
S4	O341	S3	125.24(9)	O41	S4	N2	109.57(8)
S6	O561	S5	125.50(9)	O42	S4	O341	110.64(8)
O121	S1	N1	100.51(8)	O41	S4	O42	124.59(9)
O11	S1	O121	103.53(8)	O52	S5	O561	103.75(9)
O12	S1	N1	105.18(8)	O51	S5	N1	105.51(9)
O11	S1	N1	110.15(9)	O52	S5	N1	109.93(9)
O12	S1	O121	110.57(9)	O51	S5	O561	110.75(8)
O11	S1	O12	124.5(1)	O52	S5	O51	124.88(9)
O21	S2	O121	103.18(8)	O561	S5	N1	99.12(8)
O22	S2	N2	105.36(8)	O561	S6	N2	100.10(8)
O21	S2	N2	110.07(8)	O62	S6	O561	103.25(8)
O22	S2	O121	110.09(8)	O61	S6	N2	105.27(8)
O21	S2	O22	125.45(9)	O62	S6	N2	109.55(9)
O121	S2	N2	99.85(8)	O61	S6	O561	110.44(8)
O31	S3	O341	103.73(9)	O62	S6	O61	125.46(9)
O32	S3	N1	105.31(8)				

C. Results from quantum chemical calculations

A full geometry optimization of the trimer of $S_6N_2O_{15}$ was performed within density functional theory (DFT) using the PBE0 exchange-correlation functional and a cc-pVTZ basis set.[3,4] The calculations were also used for assigning the Raman and IR frequencies. Throughout the study the TmoleX 18 (Turbomole 7.0) program package was used.[5,6]

Table S6. Coordinates for the $S_6N_2O_{15}$ anion from quantum chemical calculations.

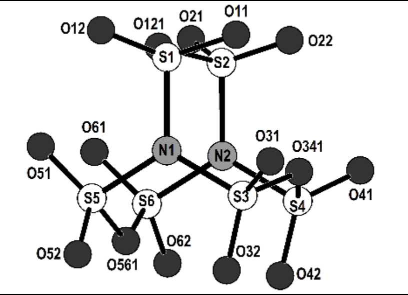

Atom	X	Y	Z
N1	0.193818	0.34328	1.429391
N2	-0.193996	-0.343335	-1.429715
O11	1.687486	2.239859	0.709318
O121	-0.55023	1.9918	-0.403916
O12	-0.468896	2.787515	1.869692
O21	-1.091974	1.682045	-2.732468
O22	1.315855	1.580856	-2.033582
O32	1.163181	-1.955135	1.78662
O31	2.653827	0.037438	2.107585
O341	2.019014	-0.581896	-0.134319
O51	-2.292478	0.702655	1.618658
O52	-1.248307	-1.167771	2.925916
O561	-1.468591	-1.409511	0.537796
O62	-1.875135	-2.272101	-1.676101
O61	-2.664416	0.045084	-1.123686
O42	0.791062	-2.61307	-0.955267
O41	2.029963	-1.067749	-2.495727
S1	0.317325	2.008392	0.989178
S2	-0.064126	1.332215	-1.825995
S3	1.60587	-0.650577	1.452743
S5	-1.350375	-0.344063	1.780691
S6	-1.732925	-1.019692	-1.034354
S4	1.224046	-1.32624	-1.36248

Table S7. Bond length [pm] for the $S_6N_2O_{15}$ from quantum chemical calculations.

Atom	Atom	Length	Atom	Atom	Length
S1	O11	141.75	S1	O121	164.12
S1	O12	141.75	S2	O121	164.2
S2	O21	141.44	S3	O341	164.14
S2	O22	141.75	S4	O341	164.12
S3	O31	141.4	S5	O561	164.13
S3	O32	141.44	S6	O561	164.13
S4	O41	141.44	N1	S1	172.64
S4	O42	141.75	N1	S3	172.67
S5	O51	141.75	N1	S5	172.67
S5	O52	141.44	N2	S2	172.67
S6	O61	141.75	N2	S4	172.69
S6	O62	141.44	N2	S6	172.69

D. Vibrational spectroscopy

IR spectroscopic data were collected with a Bruker Alpha Platinum spectrometer, using the ATR method (attenuated total reflection) in transmission mode. The spectra were measured in the bulk phase in the range from 4000 to 570 cm^{-1}. The IR spectroscopic data were processed and corrected for atmospheric influence by using the OPUS 7.5 software. Important IR bands in cm^{-1} (exptl./*calcd.*): 1512/*1535*, 1478/*1487*, 1232/*1253*, 889/*893*, 850/*831*, 699/*708*, 531/*560*, 414/*403*.

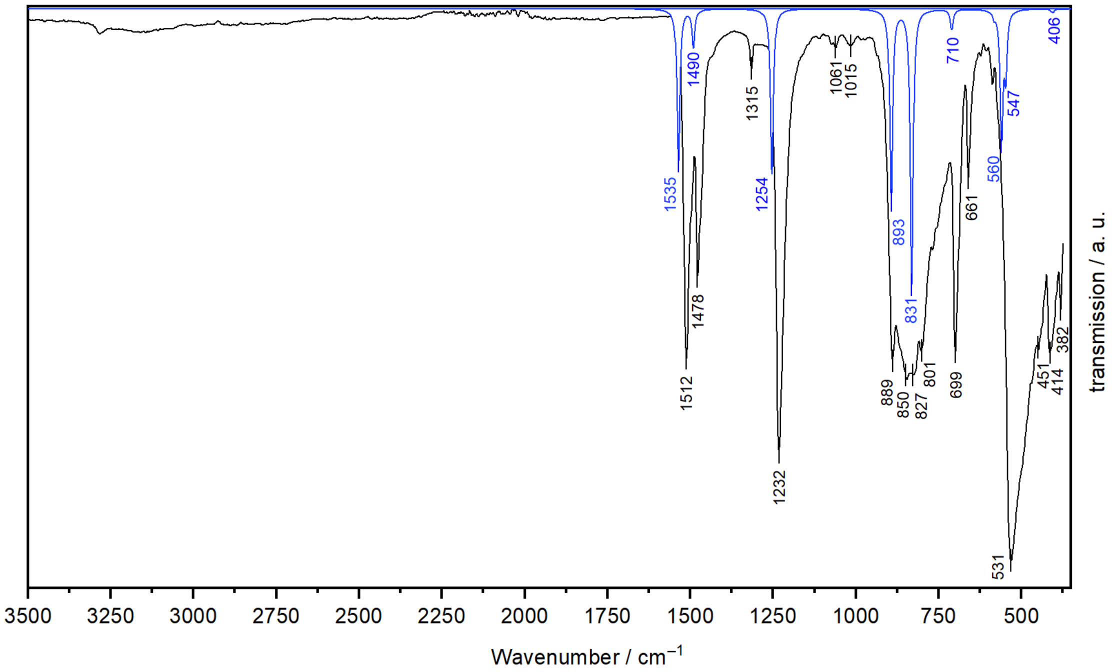

Figure S3. IR of $S_6N_2O_{15}$ compared to the respective theoretical simulations (shown in blue color).

Table S8. Experimental and theoretical (in *italics*) IR of $S_6N_2O_{15}$.

**Assignment**	**IR / cm^{-1}**
$\nu_{asym.}$ non coord. S–O	1512/*1535*,<br>1478/*1490*
$\nu_{sym.}$ S–O	1232/*1254, 1252*
$\nu_{asym.}$ NS_3	889/*893*,<br>846
$\nu_{asym.}$ S–O–S	850/*831*
$\nu_{sym.}$ S–O–S	699/*710*
δ_{asym} SO_3	531/*560*, *547, 582*
$\delta_{Sym.}$ NS_3 along N1–N2 axis	414/*406*
Not assigned modes*	1315<br>1061<br>1015

* attributed to free SO_3 according to [5]

E. Powder XRD

The finely powdered raw product was filled into glass capillaries (Hilgenberg, Ø 0.3 mm) and characterized by powder diffractometry with a STADI P powder diffractometer (Stoe, Darmstadt, Germany) using Mo-$K_{\alpha 1}$ radiation (Ge(111) monochromator and Mythen detector). The gathered data were processed with the program WinXPow (version 3.6.0.1).[6] Rietveld refinement was performed with TOPAS 5.[7] Following profile *R*-factors were received: R_{exp} = 7.26%, R_p = 5.83%, wR_p = 7.42%, GOF = 1.02.

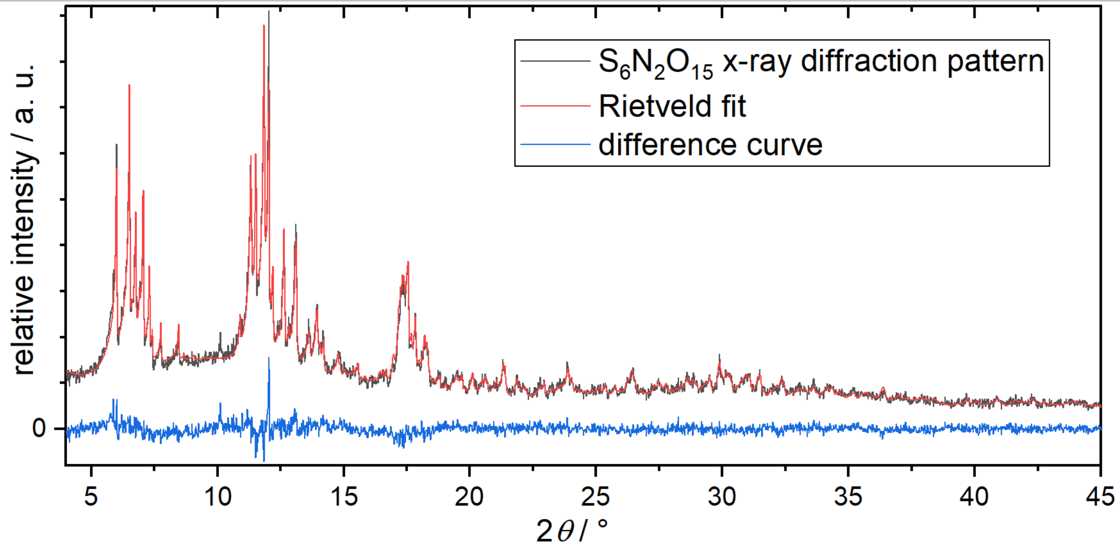

Figure S4. X-ray diffraction pattern (black), Rietveld fit (red) and difference curve (blue) from $S_6N_2O_{15}$.

References

[1] Bruker (**2018**). *Apex3*. Bruker AXS Inc., Madison, Wisconsin, USA.
[2] G. Sheldrick. *Acta Crystallogr. A* **2015**. *71*. 3–8.
[3] T. H. Dunning Jr., *J. Chem. Phys.* **1989**, *90*, 1007–1023.
[4] D. E. Woon, T. H. Dunning Jr., *J. Chem. Phys.* **1993**, *98*, 1358–1371.
[3] C. Steffen, K. Thomas, U. Huniar, A. Hellweg, O. Rubner, A. Schroer, *J. Comput. Chem.* **2010**, *31*, 2967–2970.
[4] TURBOMOLE V7.0 **2015**, *a development of University of Karlsruhe and Forschungszentrum Karlsruhe GmbH*, **1989**-**2007**, TURBOMOLE GmbH, since **2007**.
[5] A. Givan, A. Loewenschuss, C. J. Nielsen, M. Rozenberg, *J. Mol. Struct.* **2007**, *830*, 21.
[6] WinXPOW 3.6.0.1, Stoe & Cie GmbH, Darmstadt, Germany, **2018**.
[7] A. A. Coelho, *J. Appl. Cryst.* **2018**, *51*, 210–218.

Author Contributions

David van Gerven performed all of the experimental work in course of his PhD work. Mathias Wickleder is the supervisor and the leader of the project.
